# Coping Experiences: A Pathway towards Different Coping Orientations Four and Twelve Months after Myocardial Infarction—A Grounded Theory Approach

**DOI:** 10.1155/2012/674783

**Published:** 2012-12-09

**Authors:** Mari Salminen-Tuomaala, Päivi Åstedt-Kurki, Matti Rekiaro, Eija Paavilainen

**Affiliations:** ^1^Department of Nursing Science, University of Tampere, Pirkanmaa Hospital District, Science Center, Finland; ^2^Mari Salminen-Tuomaala, School of Health Care and Social Work, Seinäjoki University of Applied Sciences, Koskenalantie 17, 60220 Seinäjoki, Finland; ^3^Matti Rekiaro, Centre For Pharmacotherapy Development, The Hospital District of Southern Ostrobothnia, Central Hospital, Hanneksenrinne 7, 60220 Seinäjoki, Finland; ^4^Department of Nursing Science, University of Tampere, Etelä-Pohjanmaa Hospital District, Finland

## Abstract

*Background*. Patients recovering from a myocardial infarction (MI) are faced with a number of serious challenges. *Aim*. To create a substantive theory on myocardial infarction patients' coping as a continuum. *Methods*. Grounded theory method was used. Data were collected by using individual interviews. The informants were 28 MI patients. *Results*. The core category “coping experiences—a pathway towards different coping orientations” includes 2 main categories: “positive and negative coping experiences” (4 months after MI) and “different coping orientations” (12 months after MI). *Conclusion*. Coping with a myocardial infarction is a long-term dynamic process of dealing with varied emotions and adjustment needs. Coping is threatened, if the patient denies the seriousness of the situation, suffers from depression and emotional exhaustion, or if there are serious problems in the interaction with family members. This study stresses the importance of recognizing the patient's depressive state of mind and the psychological aspects which affect family dynamics. A more family-centered approach involving a posthospital counseling intervention is recommended. *Relevance to Clinical Practice*. The results of this study can be used in nursing care practice when organizing support interventions for myocardial infarction patients.

## 1. Introduction 

This paper presents the findings of a study that explored patients' coping experiences during the first year following a myocardial infarction (MI). Earlier studies have shown that successful coping is difficult even a year after an MI [1–6]. Effective coping following an MI calls for knowledge of the disease and of the desired effects of treatment and medication, as well as resources, a problem-solving attitude, and a sense of personal motivation [7–17]. In order to participate in all phases of MI care, patients need to be made aware of their right to participate [18].

Patients recovering from a myocardial infarction are faced with a number of serious challenges. Plenty of research has shown that besides physical suffering, patients commonly experience severe stressors and other psychological difficulties. These involve feelings of vulnerability [11, 18–21], fear of death, posttraumatic stress disorder [22–26], loss of control [22–26], anxiety [2, 3, 6, 20, 23, 27–30], and depression [31–36]. Particularly the importance of identifying incapacitating depression and anxiety has been emphasized in research [27–34, 37–41]. Depression may manifest itself as loss of energy, irritability, and reduced capacity to solve problems, and it may seriously impact on family functioning [27–34, 37–41]. Patients may also lose confidence in their bodily capabilities and functioning in work and family relationships [42, 43]. The negative effects of emotional distress on the recovery following MI make it important to study patients' coping experiences and strategies [42, 43].

Post-MI survivors attempt to make sense of what has happened in order to regain a sense of control [40]. They also have to change the patterns of their lives and action [12, 37]. It is not easy to integrate treatment-related behaviour patterns into one's everyday life or to give up old habits. The need for change is often both behavioural and psychosocial. As patients comprehend the extent of the life style changes required, they may experience further feelings of powerlessness and anxiety [40–42].

The influence the MI has on patients depends on their perception of the situation and on their coping ability [1, 40–42, 44]. Coping is defined as the constantly changing cognitive and behavioural efforts used to manage specific external or internal demands that are appraised as taxing and that may exceed the resources of the patient [45]. It includes context-specific, behavioural, and emotional processes in which the patient appraises, encounters, and recovers from contact with a stressor. It is also possible to distinguish between two types of coping strategies. Patients may use problem-focused coping strategies, in which they try to change the situation, or emotion-focused coping strategies, in which they try to regulate the emotions caused by the situation [45].

The coping process may include different stages. For example, Fleury et al. (1995), who studied women's experiences from 8 weeks up to 3 years after the MI, found the process to consist of three stages: surviving, originating, and patterning balance [46]. These stages illustrated the women's movement toward a new perspective on life and personal growth [46]. In the first stage they experienced feelings of inner chaos, isolation, and need to examine values and beliefs. It involved emotional and physical challenges that incorporated the search of personal meaning and an examination of valued relationships. Within the process of originating they created new patterns for living their lives, new expectations of self, and new ways of viewing the world. In the stage of patterning balance, their growth continued through challenges and sustained uncertainty related to MI [46]. According to Kristofferzon et al. (2008) most of the patients moved on and began to regain a balance in everyday life, but some patients experienced large difficulties with managing their everyday life and felt a lack of support from their social network [44].

Sense of coherence is another important concept in the context of coping. A sense of coherence means that one's life has worth, meaning, and purpose. It can be seen as consisting of comprehensibility, manageability, and meaningfulness [47]. Comprehensibility means that patients can make sense out of situation. Manageability means that they have resources to meet various demands. Meaningfulness refers to an individual's belief that these demands are worthy of engagement [47]. Patients' initial perceptions of illness are important determinants of different aspects of recovery after myocardial infarction. Similarly, patients' view of the future has a great impact on the strategies which they select during the coping process [7, 8, 37, 47–51].

Currently, there are not enough longitudinal studies on post-MI patients' coping and on factors affecting their coping throughout the recovery process [2, 3, 6, 13]. In this study, the aim was to create a substantive theory on MI patients' coping. For that purpose, the patients' experiences 4 and 12 months after an MI are described. Another aim was to understand the social and interaction processes during the convalescence period after the patients' discharge from hospital.

The results of the study can help nursing and medical professionals understand in what kind of issues and situations their patients most need their support. It is also important to make visible the patients who need the most support and counselling in order to achieve control over their disease. This might help to choose the appropriate interventions to promote the patients' adherence to health behaviour change [9–12, 14, 17] and to reduce psychological distress in order to improve long-term prognosis and quality of life [7, 8, 15–17].

## 2. Methods

A grounded theory methodology was selected because of its focus on the informants' personal experiences and on the identification of interaction influences. The methodology is also suitable, because a new perspective on everyday phenomena was required, both conceptually and from the viewpoint of describing the interactive process [52].

### 2.1. Sample and Data Collection

Theoretical sampling was selected in order to identify the patients' coping experiences. All the participants selected had personal experience of MI and of seeking to cope with it; they were seen as experts of their own situation. The purpose of theoretical sampling was to collect data that would maximize opportunities to develop concepts in terms of their properties and dimensions, to uncover variations, and, finally, to identify relationships between concepts. The data were collected until saturation was reached. The researcher continued selecting interviewees until they were saying nothing new about the concepts being explored [52].

The data were collected in 2006 and 2007 by using individual interviews. The informants were 28 patients, who had suffered a myocardial infarction (Table 1). The age of the patients varied from 32 to 82 years. The inclusion criteria were a first-time myocardial infarction and no cognitive or memory problems. Most patients, 25 out of 28, had had ST segment elevation myocardial infarction (STEMI), whereas 3 patients had suffered non-ST segment elevation myocardial infarction (NSTEMI). The majority of the patients, or 16, had been treated by percutaneous coronary intervention (PCI), 6 had had coronary artery bypass grafting (CABG), and 6 patients had received medication treatment for their condition. The informants had not been involved in any major rehabilitation programmes, although a few patients had participated in 2-day rehabilitation courses. Half of the patients had suffered from angina pectoris after MI, and three persons reported persistent symptoms of heart failure at 12 months following MI, whereas there had been no cases of atrial fibrillation after discharge from hospital. None of the informants had previous diagnosis of depression and none of them used psychoactive drugs.

Potential informants in one university hospital and one central hospital, from a single ward in both hospitals, were approached by cardiac nurses in a counselling situation. These nurses were either ward managers or registered nurses appointed as rehabilitation counsellors, and they were not directly involved in day-to-day patient care. Patients with no apparent cognitive deficits were given introductory letters summarizing the purpose of the study, with the assurance, both orally and in writing, that their narratives would be treated confidentially and that they could withdraw at any point. Written consent was obtained from all the participants when they were still hospitalized for acute MI. In order to ensure a good quality of selection of the participants the researcher had counselled the cardiac nurses about the content of information to give to the patients before starting the selection of the participants. In addition to oral instructions to the cardiac nurses the researcher also provided them with a written information sheet which included the purpose of the study, inclusion criteria to choose participants (a first-time myocardial infarction, no diagnosis of dementia, and no cognitive or memory problems), and ethical viewpoints to help nurses to select the participants.

The interviews were conducted by the same researcher in the patients' homes 4 and 12 months after the myocardial infarction. It was thought that by 4 months, having had their medical follow-up visit at 2 months following MI, patients would have become fully aware of their changed situation and its consequences. The interviews lasted 60–90 minutes, with the mean length of 75 minutes. The interview questions, based on the study aim, were flexible, so as not to constrain the theory development [52]. Each interview was opened with the question, “Please tell me about your coping with myocardial infarction,” and the interviewees were asked to freely describe their experiences. Each individual interview brought depth and helped to further develop the subsequent interview themes [52]. The researcher audio-taped and transcribed the data on computer by permission of the participants. The data amounted to 640 double-spaced pages altogether, with the mean length of transcript being 12 pages.

### 2.2. Data Analysis

The two sets of data, gathered 4 months and 12 months after the patients' MI, were analyzed separately using the grounded theory techniques [52]. The transcripts were examined line by line for emerging themes, and these themes were used to inform further data collection and analysis. The data were analyzed by using constant comparison. The questions and comparisons evolved during the data analysis guided the theoretical sampling, which was continued until saturation was reached. The analysis was based on Corbin and Strauss's inductive methodology of three coding phases [52].

Open coding began by listening to tape-recorded interviews and by reading transcripts to ensure the accuracy of the text. At the first level of coding, the data were analyzed line by line picking out all the original expressions concerning the patients' experiences of coping with acute myocardial infarction. This coding was guided by questions “What is happening and being expressed here, what does it mean, what is essential and in what context are the interaction and other action taking place?” The coding process led to segmentation of the data into smaller codes, which were labeled according to their meaning. Data were compared with data to find similarities and differences. The open codes helped to separate data and to see relevant processes, and the coding helped to focus further data collection [52].

Similarities were identified in original expressions, which were combined into empirical codes. These were formed into substantive codes, which were divided into subcategories according to code properties [52]. 11 subcategories were formed. At the stage of axial coding, the properties and dimensions of the contents of each subcategory were examined. The subcategories with similar properties and dimensions of contents were combined into 2 main categories describing concepts of the patients' coping experiences. 

**Figure 1:**
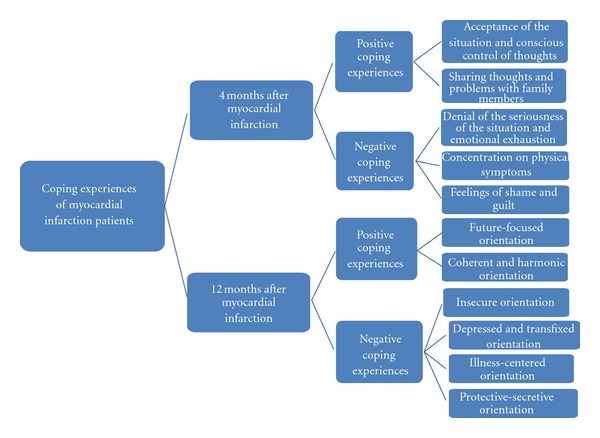
Different coping pathways towards different coping orientations.

The analysis proceeded through to the selective stage of producing a core category, which combined all concepts created up to that point. This category was labeled “coping experiences—towards different coping orientations.” The concepts describing the elements in the patients' coping between the early recovery phase (4 months) and later recovering phase (12 months) give new information about the phases of the coping process (Figure 1). 

### 2.3. Ethical Issues

The study conforms with the principles outlined in the Declaration of Helsinki [53]. Participation in the study was voluntary and based on the informants' informed consent. Confidentiality and possibility to withdraw at any time were emphasized and special attention paid to the protection of the patients' psychological and physical condition. This report does not include any private data that identify the participants. 

## 3. Results

The core category “coping experiences—a pathway towards different coping orientations” describes the participants' experiences of coping with myocardial infarction as a continuum. The social and interaction processes during the convalescence period emerged from the data that explain participants' coping as different pathways. There are causal conditions and contextual and intervening conditions related to the coping experiences 4 months after MI and different coping orientations 12 months after MI. The causal conditions include MI as a disease, its prognosis, and treatment. The contextual conditions include events and participants' emotional, social, and physical environments 4 and 12 months after MI. The intervening conditions are general conditions that have a bearing on different action and interactional strategies. The intervening conditions may be due to the patient-dependent or the family-dependent characteristics. Family dynamics has an essential meaning for emerging of different coping orientations. 

The following description of the patients' coping experiences 4 months and 12 months after the cardiac infarction presents two main categories. The main category “positive and negative coping experiences” describes the patients' experiences 4 months after the cardiac infarction and the main category “different coping orientations” their experiences 12 months after cardiac infarction.

Positive coping means that the person has accepted the situation and is ready to continue life, whereas negative coping refers to psychological immobilisation, not being able to move on or to accept and deal with the seriousness of the situation, often combined with continued focus on physical symptoms. The main category “positive and negative coping experiences” 4 months after MI was found to include two positive subcategories: firstly, acceptance of the situation and conscious control of thoughts and secondly, sharing thoughts and problems with family members. It also included three negative subcategories: denial of the seriousness of the situation and emotional exhaustion, concentration on physical symptoms, and feelings of shame and guilt. 

In the main category, “different coping orientations,” the analysis of the second set of data (12 months after MI) resulted in the positive subcategories future-focused orientation and coherent and harmonic orientation. It also included four negative subcategories: insecure orientation; depressed, transfixed orientation; illness-centered orientation; protective-secretive orientation.

### 3.1. Positive and Negative Coping Experiences 4 Months after the Myocardial Infarction

The patient interviews revealed that the seriousness of a lived-through myocardial infarction often does take not shape until 3 or 4 months after the acute situation. The participants in this study reported that the short hospital stay did not allow assimilation of all the information concerning life changes and follow-up treatment at home, and they hardly had the energy to concentrate on future. The participants described their confusion and tiredness at home after discharge. Only after some weeks did they begin to awake to reality. A 58-year-old working male described his situation as follows:

In the hospital I tried so hard to cope with the situation that I did not realize the seriousness of the situation. When I came home I awoke to reality and realized how close I had come to dying.

### 3.2. Positive Coping Experiences

#### 3.2.1. Acceptance of the Situation and Conscious Control of Thoughts

Some participants described their acute myocardial infarction as a situation, which simply had to be encountered and accepted. They considered the infarction either their destiny or a part of normal life, something that could happen to anyone and had to be lived through. Some participants spoke of the MI as having a positive impact on their life, because it had made them reflect on their values and on the meaning of life from a new perspective. In some families, the disease had brought the family members closer to one another. The majority of the participants in this study regarded it as essential to accept the situation sooner or later.

It seems that the majority of the participants interviewed for this study were not willing to give up. Many of them described the illness as a life situation, which could cause depression and hopelessness, unless they deliberately maintained a positive attitude to life. By an optimistic attitude they meant serenity, trust, and perseverance. These participants felt that they were able to affect their life course. They referred to their zest for life and happy mood and remained positive and hopeful. The time directly after the MI was understood as a resting time, suitable for renewing physical and emotional resources and for preparing for a future of good and happy things. These participants experienced that they had the possibilities and resources required to achieve their various goals. They also developed dreams regarding their future.

Many participants' serenity seems to be based on a long life experience of coping with trials and troubles. Having earlier undergone difficult situations or diseases, they were able to rely on repeating their positive coping experiences. These interviewees evidently retained their mental balance and courage for life during convalescence without major emotional turmoil, continuing their normal life throughout the sick leave.

The majority of the participants in this subcategory, especially men, considered it very important to control and manage their thoughts consciously. They tried to forget their pains and other symptoms by concentrating on pleasant and comfortable ideas. There was deliberate avoidance of thinking of MI in terms of illness. These persons did not deny the seriousness of the situation, but they made a conscious choice to concentrate their thoughts on more positive aspects of life.

Besides seeking to control their emotions by means of positive thoughts, the participants concentrated on solving problems in a logical way. They felt that they managed their own life and should not try to accommodate themselves to anybody's overprotectiveness.

#### 3.2.2. Sharing Thoughts and Problems with Family Members

All patients in this study received some degree of social support from their families; 3 persons mentioned friends as their most significant source of support. The support from the family facilitated the participants' coping by enhancing their trust in their ability to deal with the illness and recovery time at home. Compassion and understanding were forthcoming; the participants reported that they were able to share their burdens.

### 3.3. Negative Coping Experiences

#### 3.3.1. Denial of the Seriousness of the Situation and Emotional Exhaustion

Some participants interviewed for this study were inclined to deny the seriousness of the situation. They tried to avoid thinking about the disease and its symptoms, concentrating on current news and events instead. More men used gallous humor in order to relieve their stress. Denial can also seen as an essential way to cope with a difficult situation. It gives time and room to gather one's psychological resources to adjust to the situation.

Four months after the MI, many of the participants still felt physically and emotionally drained. They explained how their everyday lives were characterized by lack of resources. Some of the participants interviewed tried hard not to think about the MI, ignoring physical symptoms and shunning emotions related to their situation. Some of them reported severe tiredness and exhaustion four months after the MI, having struggled to endure and control insecurity in order to protect their family members.

The emotional exhaustion of these participants manifested itself in depressive and hopeless thoughts, circling around themselves. They did not trust their ability to return to normal life, thinking that they would have to give up work or planning for future. Some of them felt that they had failed in their life. In patients representing this subcategory, the exhaustion was not accompanied by severe physical symptoms.

#### 3.3.2. Concentration on Physical Symptoms

Many participants in this study experienced that they had not recovered well from the MI, because they still suffered from angina pectoris, arrhythmia, and dyspnea. The presence of these symptoms induced fear of recurrence of the infarction. The life of these men and women concentrated on their physical symptoms. They explained that the symptoms controlled their everyday life; they were conscious of their physical condition all the time. This was experienced as very stressful.

#### 3.3.3. Feelings of Shame and Guilt

Many of the working-aged men and women interviewed for this study found it shameful to stay at home during their convalescence. They would have preferred to work and participate in normal social life. Being idle was particularly difficult for men, who considered themselves as the family breadwinners. They felt that they had let down their spouses and experienced guilt, seeing their spouses attending to all the housework after full days at work. There were also concerns that the neighbors might regard the convalescents as lazy work evaders. A few participants expressed their feeling that the MI might diminish their value as citizens, as they would not be able to contribute during their sick leave.

Most of the men were also irritated at what they considered their spouses' overprotectiveness. They felt that the wives kept an eye on them all the time and that they were not trusted anymore. Women reported that they would have liked to contribute more to housework, but their spouses would not allow them to do that. Both men and women experienced that they had lost a part of their independence and personal space, being under the spouse's controlling eye.

Some men experienced shame, because their employers did not understand the seriousness of the illness, but demanded their return to work as soon as possible. This was a situation with conflicting interests to the men, who were willing but unable to resume work.

### 3.4. Different Coping Orientations 12 Months after the Myocardial Infarction

In the analysis of the second set of data, which describes experiences 12 months after the MI, the participants' positive coping experiences were found to involve a future-focused orientation and a coherent and harmonic orientation. Finally, there were four subcategories of negative coping experiences: an insecure orientation; a depressed, transfixed orientation; an illness-centered orientation; a protective-secretive orientation.

#### 3.4.1. Future-Focused Orientation

Most of those men and women, who expressed a positive attitude to future, were still in working life. Their thoughts seemed to be very much focused on future. Half of all the interviewees believed, for example, that it was important that they took responsibility for their own treatment, medication, and lifestyle changes. Many of them had acquired knowledge about the disease, making an effort to prevent complications and recurrence. As far as their future and health were considered, they considered themselves the responsible key persons. These participants also recognized and discovered new challenges and possibilities. They explained that, having by that point dealt with some difficult emotions and fears regarding MI, they now were able to trust their coping abilities. It was typical of these participants that they had been able to accept the situation and discuss it with their families already 4 months after the infarction.

#### 3.4.2. Coherent and Harmonic Orientation

Most of those participants, whose attitude to life was one of serenity and harmony, were elderly. One third of all the participants said that their life was balanced 12 months after the MI. They declared that their life had a meaning, it was harmonic, and they had reached an equilibrium of emotions. These men and women had confidence in life tomorrow, belief in God, or trust in their own capabilities to control their lives. For them, coping meant mastering one's feelings when possible and accepting the inevitable when not. Some of them had been compelled to cope with significant loss earlier in their lives, and they had managed to do so without getting depressed.

All the participants with this orientation had accepted their situation already 4 months after the infarction, and their life at 12 months after the MI made an impression of a serene, harmonic continuum. The participants in this group had also felt supported by their family members.

Many participants in this study emphasized how important it was to be able to return to everyday life and enjoy routines and habits. This was a way to reduce stress and worrying. Their experiences revealed the importance of concentrating on positive and empowering things, instead of occupying oneself with illness, symptoms, and limitations. They also said that coping meant breaking problems into manageable bits and working them through one at a time.

#### 3.4.3. Insecure Orientation

Some of the participants interviewed for this study experienced that they did not have adequate information about their followup and self-care even 12 months after the myocardial infarction. They declared that they would have needed clearer guidelines concerning the rehabilitation process. The follow-up appointments with medical or nursing staff had stopped at 2 months after the MI, and the participants felt left to fend for themselves.

These participants were anxious because they were not certain how much they were allowed to stress their body; they lacked the proper insight on the limits of their physical condition. They explained that they did not know when and how to increase physical activity and when to resume their normal lives. They were also uncertain about how to continue their medical treatment. Furthermore, the participants indicated that they did not always recognize their own resources on a concrete and experiential level.

#### 3.4.4. Depressed, Transfixed Orientation

In this study, there were also some participants, who felt emotionally and physically powerless and described their life as “transfixed.” They did not have the will, desire, or psychological power to deal with or change their situation. Lacking the courage to participate in social happenings, they stayed at homes. Their everyday life was apparently characterized by varied fears, anxiety, and unhappy thoughts. The interviewees, who seemed depressed, explained that they often experienced a fear of death or hoped for death and wanted to give up everything. They also felt anxious if they were compelled to concentrate on new or strength-demanding issues. Three of the working-aged participants in this study, one woman and two men, reported feeling so hopeless, depressed, and powerless that they did not think they would return to work.

#### 3.4.5. Illness-Centered Orientation

In some cases, the experienced MI had apparently become the centre of the participants' life. Some of the men and women concentrated on their symptoms and waiting for the recurrence of the MI. They felt paralyzed or completely incapacitated and very dependent on their family members. Those especially, who suffered from heart failure as a result of the infarction, seemed to have adopted a strong illness-centered orientation towards life.

A woman of 82 years woman reported how her life had been centered around her heart disease. Since the infarction, she had suffered from chest pain and shortness of breath almost daily. The symptoms were so severe that she was unable to concentrate on anything else.

Sometimes illness becomes a means to gain more attention from family members, resulting in the family's activities becoming centered around the participant's illness.

A symptom can also represent a concrete sign of life for the patient—because they can still feel the symptom.

#### 3.4.6. Protective-Secretive Orientation 

It was very common even 12 months after the myocardial infarction that the men and women experienced a strong need to protect their family members from the seriousness of their illness. Over 50% of them tried to hide their symptoms and did not discuss them with their spouses. Sometimes this seemed to result in interaction problems and lack of confidence and trust:

I am trying to hide my pain from the wife, but I think she suspects that I do not tell everything. I do not want to make her worry, on the other hand this hiding may damage our mutual interaction, we are not so forthright and open like we used to be.

## 4. Discussion 

The results of this study reveal great differences in patients' dynamic and individual coping processes after a myocardial infarction. The comparison of the same participants' situation 4 and 12 months after the MI reveals what can be described as a continuum of coping. The participants, who had accepted the reality of having suffered a cardiac infarction and had dealt with their subsequent emotions and fears by 4 months after the MI, were balanced and serene also at 12 months. Their coping can be seen as representing either a future-focused or a coherent and harmonic orientation. The future-focused orientation is, perhaps naturally, more typical of patients who are still active in working life. The sense of coherence, as shown by Antonovsky (1993) [47], an internal locus of control, and sharing thoughts and problems with family members (social support) seem to guide the direction of this kind of positive coping processes. Adopting health-promoting behaviour is also dependent on the patient's self-efficacy, sense of coherence, and motivation and readiness to change.

In contrast to the patients described above, the interviewees, who were anxious or depressed or closely observed their symptoms four months after their infarction, still represented a depressed, transfixed, or illness-centered orientation at 12 months. These patients had less physical and emotional resources to deal with their changed situation, and they did not rely on their coping with the MI. Many of them denied the seriousness of the disease or suffered from various fears, shame, and guilt. These men and women had a tendency to observe their physical symptoms and to anticipate another infarction. There was no evident association between the type of MI and coping orientation.

An important finding of this study was that the patients did not have time to become fully aware of their new situation during the short hospital stay, which makes it demanding for the medical and nursing staff to identify patients prone to depression or denial. Awakening to reality 2–4 months after their discharge, the patients would then need support from nursing staff and doctors in their coping. Fleury et al.'s study (1995) has also shown that the coping process may include individual questioning, patterning, feedback, and repatterning that leads to balance over time [46]. According to our study it is not always possible to achieve a balance even 12 months after MI.

Another problem calling for attention concerns the interaction between patients and their family members. Even after a year following the infarction, many patients seem to conceal their situation to protect their families or find it difficult to discuss their illness because of experienced guilt or shame. Spouses, on the other hand, are often aware that everything is not in order and may become overprotective, inadvertently increasing the patients' sense of inferiority or feeling that they are not relied on anymore. The lack of open communication can thus increase stress and lead to further emotional problems. In this study, these patients' attitude at 12 months was termed “protective-secretive orientation.” The findings are similar to the results of earlier studies. For example, Svedlund and Axelsson (2000) found that it is typical of myocardial infarction patients to feel ashamed for being weak [15]. Earlier studies have also proposed that spouses' overprotectiveness can reduce the patient's independence and cause tension between family members [5].

The study did not produce much knowledge about whether the work of nursing staff influenced the patients' coping in a significant manner. This does not mean that the possibility of such an influence could be excluded.

Based on the results, a nurse-led counseling and discussion intervention is recommended for MI patients approximately 4 months following the infarction. At this point it would be easier to identify those patients, who still deny the seriousness of the situation, do not know how to deal with the disease-related issues or emotions, or suffer from depression and emotional exhaustion. The discussion would also provide an opportunity to help the patients become conscious of their individual disease control and life management resources.

Another recommendation based on the findings is a more family-centered approach to nursing patients with MI. The family should be involved at an early stage, already in hospital, to help family members support the patient's coping. It is equally important to engage the family in the suggested counseling and discussion interventions at 4 months.

## 5. Conclusion

Coping with a myocardial infarction is a long-term dynamic process of dealing with varied emotions and adjustment needs. Coping is threatened, if the patient denies the seriousness of the situation, suffers from depression and emotional exhaustion, or if there are serious problems in the interaction with family members. This study stresses the importance of recognizing the patient's depressive state of mind and the psychological aspects which affect family dynamics. It is important to arrange an updating education for all health care personnel on the recognition of patients' depressive symptoms. It would be important to arrange meetings with counseling nurses specialized in myocardial infarction 4 months after the myocardial infarction. During this meeting the nurse could give empowering counseling for the patient and try to find out if the patient still suffers from denying the seriousness of the situation or depression. These empowering counseling meetings could be arranged in the hospital, and it would be important to assess during these meetings whether the patient also needs the psychiatric help. It would be important to take the patient's spouse along to these meetings. A more family-centered approach involving a posthospital counseling intervention is recommended.

## Figures and Tables

**Table 1:** Characteristics of the study participants.

Characteristics of the participants	
Characteristic	Number
Gender	
Male	16
Female	12
Age	
Mean	65
Range	32–82
Type of MI	
STEMI	25
NSTEMI	3
Treatment procedures	
PCI	16
CABG	6
Medication	6
